# Pregnancy during Adolescence and Associated Risks: An 8-Year Hospital-Based Cohort Study (2007–2014) in Romania, the Country with the Highest Rate of Teenage Pregnancy in Europe

**DOI:** 10.1155/2017/9205016

**Published:** 2017-01-04

**Authors:** Demetra-Gabriela Socolov, Magdalena Iorga, Alexandru Carauleanu, Ciprian Ilea, Iolanda Blidaru, Lucian Boiculese, Razvan-Vladimir Socolov

**Affiliations:** ^1^Obstetrics and Gynecology, University of Medicine and Pharmacy “Gr. T. Popa” of Iasi and University Maternity Hospital “Cuza Voda”, Iasi, Romania; ^2^Behavioral Sciences Department, University of Medicine and Pharmacy “Gr. T. Popa” of Iasi and Saint Mary University Child Hospital of Iasi, Iasi, Romania; ^3^Department of Statistics, University of Medicine and Pharmacy “Gr. T. Popa” of Iasi, Iasi, Romania; ^4^Obstetrics and Gynecology, University of Medicine and Pharmacy “Gr. T. Popa” Iasi, Iasi, Romania

## Abstract

*Aim*. To determine pregnancy and delivery outcomes among teenagers.* Materials and Methods*. An 8-year retrospective comparative hospital-based cohort study is analysing singleton pregnancy comorbidities and delivery parameters of a teenage group under the age of 20 compared with a young adult group 20–24 years of age in a university hospital.* Results*. Teenage is a risk factor for preterm birth <37 weeks (1.21 [1.08–1.35]), foetal growth restriction (1.34 [1.21–1.48]), episiotomy (1.27 [1.21–1.34]), uterine revision (1.15 [1.06–1.25]), APGAR <7 at 1 min (2.42 [1.21–1.67]), cephalopelvic disproportion (1.26 [1.07–1.48]), and postpartum haemorrhage (1.42 [1.25–1.62]); however, caesarean delivery occurs less frequently in teenagers than in adults (0.75 [0.70–0.80]). The following comorbidities are risk factors for teenage pregnancy (risk ratio [CI 95%]): anaemia (1.13 [1.10–1.17]), low urinary tract infection (1.10 [1.03–1.18]), pediculosis (2.42 [1.90–3.00]), anogenital condyloma (1.50 [1.04–2.17]), and trichomoniasis (1.74 [1.12–2.68]). The risks for hepatitis B and hepatitis C, premature rupture of membranes, and placenta praevia were lower compared with those in the young adult group, respectively, 0.43 (0.26–0.71), 0.90 (0.85–0.96), and 0.29 (0.20–0.41), while the risk for gestational diabetes and preeclampsia were the same in both groups.* Conclusion*. Considering the high risks for teenage pregnancy, this information should be provided to pregnant adolescent women and their caregivers.

## 1. Introduction

The World Health Organization (WHO) defines teenage pregnancy as pregnancy in which the mother is under the age of 20 at the time the pregnancy ends [1]. About 16 million girls 15 to 19 years old and another 1 million girls under 15 give birth every year, most in low- and middle-income countries.

Complications occurring during pregnancy and labour represent the second most frequent cause of death in teenagers 15–19 years of age. The babies born to this age group are at a higher risk of dying than those born to women 20 to 24 years of age [1].

In the study of Sedgh et al., based on United Nations Statistics Division's Demographic Yearbook data [2], Romania reported in 2011 around 34,700 teen pregnancies among females 15–19 years of age. With a pregnancy rate (defined as number of pregnancies per 1,000 females 15–19 years of age) of 61% and a birth rate (defined as number of births per 1,000 females 15–19 years of age) of 35%, Romania has the highest rates in Europe. For the age category of 10–14 years of age, the rate of pregnancies (defined as number of pregnancies per 1,000 females 10–14 years old) was 2.64%_,_ and the rate of births (defined as number of births per 1,000 females 10–14 years old) was 1.40%, the highest in the world according to these statistics.

The socioeconomic factors are important for the prognosis of teenage pregnancies, as has been shown in a previous article published by this team [3].

The aim of this study was to compare pregnancy and delivery outcomes among adolescent women with those of young adults in a tertiary maternity centre, by focusing on the influence of medical factors.

## 2. Materials and Methods

This is a retrospective comparative hospital-based cohort study of all singleton pregnancies and deliveries that occurred in a teenage group (<20 years old) compared with a young adult group (aged 20–24 years old) at “Cuza Voda” Hospital, Iasi, Romania, which is the reference centre for the region of northeastern Romania. Inclusion criteria for all cases and controls were singleton pregnancy, age < 25 years, gestational age at delivery > 24 weeks, and delivery between January 1, 2007, and December 31, 2014. The ethics committee of the hospital approved this study. We extracted data from the hospital's electronic database for the following two parameters: pregnancy comorbidities and parameters concerning delivery, using the International Classification of Diseases, 10th Revision (ICD-10) diagnostic codes.

We compared obstetric characteristics of the two age groups, analysing the frequency of data by using the chi-square (*χ*^2^) and Fischer exact tests for expected frequencies <5. We evaluated pregnancy and delivery outcomes for most maternal and neonatal outcomes, estimating the risk ratios (RRs) and 95% confidence intervals (CIs). *p* < 0.05 indicated statistical significance.

We repeated all these comparisons after splitting the teenage group into two groups (12–17 years and 18-19 years), and we compared both subgroups of teens with the control group (20–24 years of age).

All analyses were conducted using SPSS 17.0 (IBM, Armonk, New York, USA), and data were charted using Microsoft Excel for Windows 2010.

## 3. Results

A total of 48,308 deliveries were registered between January 2007 and December 2014 at the University Maternity Hospital “Cuza Voda.” A small number of them (*n* = 3,961, 8.19%) involved teenagers (<20 years of age).

Of the total deliveries, only singleton pregnancies (*n* = 46,930) were included in this study. The singleton pregnancies were divided into two groups: pregnant teenage women (*n* = 3,891, 8.3%) and, as the control group, pregnant women 20–24 years old (*n* = 9,479, 20%).

The age of the study group patients ranged from 12 to 19 years, with a mean age of 17.81 ± 1.25 years, while the age of the control group ranged from 20 to 24 years, with a mean age of 22.18 ± 1.42. The number of deliveries increased with age: 12-year-olds having 4 deliveries and the 19-year-olds having 1,450 (Table 1).

### 3.1. Comorbidities

Registered comorbidities are presented in Table 2. The teenage group had no risk for either chronic or gestational hypertension (*p* = 0.65) or for preeclampsia (*p* = 0.45). The number of cases of eclampsia was too small to interpret.

Foetal anomalies detected at birth did not occur more frequently in the teenage group (*p* = 0.18). Instead, the incidence of anaemia and lower urinary tract infections was higher in adolescents compared with that in young adults (*p* < 0.01 and *p* < 0.02, resp.). Diabetes and obesity were more common in young adults than in the teenage group (*p* = 0.01 and *p* = 0.02, resp.).

Regarding infections associated with pregnancy, pediculosis occurred more frequently in the teenagers than in the control group (*p* < 0.01), as did anogenital warts (*p* = 0.03) and trichomoniasis (*p* = 0.02). Hepatitis B and hepatitis C were more common in adults (*p* < 0.01). Premature rupture of membranes and placenta previa occurred more frequently in teenagers ((0.90 [0.85–0.96], *p* = 0.03) and (0.29 [0.20–0.41], *p* < 0.01), resp.). We could not establish a correlation between the presence of chorioamnionitis and patient age (*p* = 0.27).

### 3.2. Delivery Characteristics

Concerning delivery characteristics, teenagers represent a risk group for preterm birth. But the higher the prematurity, the lower the correlation with parturient age. We noticed *p* < 0.01 for births < 37 weeks, *p* = 0.01 for births < 34 weeks, and no correlation, *p* = 0.40, for births < 28 weeks (Table 3).

The birth weight of the foetuses is also affected by the age of the mother. A good correlation exists between low birth weight < 2500 g and maternal age < 20 years (*p* < 0.01), but this correlation does not exist for very low birth weight foetuses < 1,500 g (*p* = 0.35). We also found a correlation between foetal growth restriction and teenage age at delivery (*p* < 0.01).

Caesarean delivery was more common in the adult group than in the adolescent group (*p* < 0.01). On the other hand, episiotomy was more common in adolescents (*p* < 0.01).

Perineal ruptures of 3rd and 4th degrees, cervical lacerations, and instrumental deliveries with forceps and vacuum extractor had no statistical correlation with a particular age group (*p* = 0.62, *p* = 0.05, and *p* = 0.33). Cephalopelvic disproportion and breech presentation were more common, as expected, in the teenage group (*p* < 0.01 and *p* = 0.03, resp.), but humeral presentation (*p* = 0.28) did not appear more frequently in a certain age group.

Postpartum haemorrhage as well as uterine revision proved to be more common in the teenage group (*p* < 0.01).

Adolescent births had more cases of Apgar < 7 at 1 minute than adult births had (*p* < 0.01). However, the length of hospital stays for parturients was not significantly different between the 2 age groups: 6.44 ± 3.89 days for teenagers versus 6.59 ± 4.23 days for young adults (*p* = 0.06).

## 4. Discussions

Our hospital is a university tertiary unit, the reference centre for the northeastern region of Romania, a region with a low socioeconomic level and with a high overall birth rate, with 48,308 births between 2007 and 2014, representing 2.92% of the national birth rate in the same period [4]. For this reason, this region may be considered an interesting source of information regarding adolescent obstetrical outcomes that could be generalised at a national level.

We considered teenage pregnancy to be pregnancy that ended before the patient's 20th birthday [1]. We considered 20–24-year-old parturients as the control group, which is reported also in these articles: WHO [1], Nove et al. [5], Sedgh et al. [2], and Gortzak-Uzan et al. [6]. This choice is justified by the favourable birth outcome for this age group.

Pregnancy comorbidities, chronic and gestational hypertension, and preeclampsia and eclampsia, occurred similarly across age groups. These results support the findings of Torvie et al. [7], Traisrisilp et al. [8], Dedecker et al. [9], Gortzak-Uzan et al. [6], and Gupta et al. [10]; however, other authors have reported high incidences of eclampsia in teens [11–13].

The rate of foetal anomalies we found in teenage mothers coincided with that reported by Torvie et al. [7] and Sagili et al. [14]; however, Shrim et al. [15] reported higher rates of congenital anomalies in the babies of teenage mothers.

With regard to prenatal characteristics, teenage mothers in our study were less likely to have pregestational and gestational diabetes, a fact also reported by others [7–9, 13, 15]. Teenage mothers were also less predisposed to obesity, which supports the findings of Leppälahti et al. [13].

Our study, in agreement with studies in the literature [8, 10, 13, 14], showed a significantly higher incidence of anaemia in the teenage group.

Alouini et al. [16], Leppälahti et al. [13], and Gupta et al. [10] found that low urinary tract infections are more frequent in pregnant teens than in pregnant adults. Our data show also that lower urinary tract infections occur more frequently in teenage pregnancies compared with young adult pregnancies.

Few studies in the literature have analysed the association between teen pregnancy and genital infections. Hidalgo et al. [12] found no differences in the frequency of genital infection with* Gardnerella vaginalis* and Human Papilloma virus, but the incidence of trichomoniasis and candidiasis among teenagers was significantly higher.

In our study, with the exception of genital warts and trichomoniasis, we could not find an association between teen pregnancy and bacterial vaginosis,* Candida*, or syphilis. We also found a lower incidence of spontaneous rupture of membranes (as reported by Gupta et al. [10] and Sagili et al. [14]), whereas the incidence of chorioamnionitis in the teenage group was not significantly different from that in the young adults (*p* = 0.27). In turn, Shah et al. [17] reported chorioamnionitis to be three times more frequent in teenagers.

Infections with hepatitis B and hepatitis C viruses were higher in the control group, as reported by Kurth et al. [18].

Our results are in accordance with almost all studies that found higher risks of preterm birth [6, 8, 16, 19], low birth weight [8, 15, 19, 20], and foetal growth restriction [7, 8, 15, 19].

Shrim et al. [15] reported that, in young women under the age of 20, there was a greater than threefold increased risk of delivery before 34 weeks and over a fourfold increased risk for delivery below 28 weeks compared with deliveries in older mothers. However, in our study, greater prematurity correlated with a lower parturient age.

The same consideration is applicable to the birth weight of foetuses. There is a good correlation between low birth weight < 2,500 g and maternal age < 20 years, but this correlation does not exist for very low birth weight foetuses < 1,500 g.

Concerning the modality of labour, our findings suggest a lower risk of caesarean and operative vaginal delivery in women < 20 years of age compared with those between 20 and 24 years of age.

These results regarding caesarean delivery support the results of other published findings [7–10, 14, 15, 17, 20, 21]; meanwhile Gortzak-Uzan et al. [6] found no difference between the two groups, and Malabarey et al. [22] reported elevated risk of caesarean delivery in the 12–14 years group compared with the 15–20 years age group.

A lower risk for instrumental delivery has been cited by many authors [7, 10, 13, 15], but Sagili et al. [14] found no statistical difference between the two groups, and Shah et al. [17] found the rate of instrumental delivery significantly higher among the teenage group.

Concerning postpartum haemorrhage and manual/instrumental uterine revision, we found a higher incidence in the teenage group ((1.42 [1.25–1.62], *p* < 0.01) and (1.28 [1.17–1.40], *p* < 0.01)). Sagili et al. [14], Shah et al. [17], Traisrisilp et al. [8], and Leppälahti et al. [13] did not find a significant difference between both groups.

We did not find a higher risk in our study for either cervical or perineal lacerations or for perineal rupture of 3rd and 4th degrees, but episiotomies were more common in the teenage group.

Torvie et al. [7] also found a decreased risk of 3rd- and 4th-degree perineal rupture, but other studies report more perineal lacerations and episiotomies [14, 16].

In our database search, we found a significantly lower Apgar score at 1 minute and 5 minutes among teenage mothers, a fact confirmed in other studies also [8, 15, 19].

### 4.1. The Novelty of the Study

Even if Romania holds second place in WHO statistics regarding teenage pregnancy, this study is the second study published in the English literature about medical aspects related to teenage pregnancy and delivery outcomes in Romania.

In 2015, a study was conducted in a different geographic region of the country (the central one) covering a shorter period of time (2011-2012) with fewer teenage pregnancies (*n* = 395) and fewer adult controls (*n* = 736) [23]. It focused particularly on social factors in teenage births. Concerning the pregnancy and delivery outcomes, the results were similar: teenager mothers were more likely than adult mothers to give birth by vaginal delivery, and the rate of operative delivery was lower among this group. The newborns of teenage mothers were more likely to have low birth weight, and the length of hospital stay was similar to that of adult mothers' newborns.

Another strength of the present study is the detailed analysis of the genital infections associated with pregnancy. One of the mechanisms for preterm birth is the high frequency of genital infections enhancing local prostaglandin levels: few studies have examined the infections associated with pregnancy [15, 24]. In our hospital, performing a cervical culture is mandatory for all pregnant women at admission, and lochio culture is performed in the first 48 hours after birth. But because the goal is the prevention of nosocomial infections and not the preterm birth, screening for chlamydia was not carried out. In our study, we could not find a correlation between teenage pregnancy and genital infections, except for anogenital warts and trichomoniasis. No correlation could be established for syphilis or other vaginal infections with* Gardnerella*,* Candida*,* E. coli*,* Streptococcus*,* Staphylococcus*, or* Klebsiella*. Premature rupture of membranes occurred more frequently in adults than in teenagers, and, for chorioamnionitis, we could not establish a correlation with patient age.

The other mechanisms described by Shrim et al. [15] for preterm birth, that is, low birth weight and being small for gestational age, are related to the gynaecologic immaturity of the teenager who is less than two years after menarche and has a relatively less well-developed uterine and cervical blood supply. Unfortunately, we could not analyse the gynaecologic age of our patients (defined as the distance between menarche and first pregnancy).

This research has some limitations. First, this is a relatively small sample size of early adolescence (<15 years old) (*n* = 50) to be able to stratify the adolescent pregnancy and delivery outcomes on subgroups by age, to better evaluate the effect of mother's age on pregnancy and birth finality. Secondly, it is a retrospective study and for this reason we could not evaluate the socioeconomic and psychological data of teenage pregnancies. Another limitation of the study is that testing for genital infection by chlamydia, as a potential risk factor of preterm birth, was not performed.

Most of the patients were from rural areas. Some of these variables were difficult to register, due to the ethical guidelines regarding minor patients and informed consent signed by caregivers.

The Center for Disease Control and Prevention [25] recommends splitting the adolescent group (<20 years old) into three groups: <15 (young adolescents), 16-17 (young teens), and 18-19 (older teens). We split our group into only two categories (<18 and 18-19), because we had a very low number of young adolescents (50 teens under age of 15), which could bias the results.

## 5. Conclusions

Our study confirms prior findings that infants born by teens are at higher risk of preterm delivery, low birth weight, FGR (foetal growth restriction), and foetal distress.

Our data about delivery may reassure obstetricians that teen births have a caesarean delivery and an instrumental birth risk lower than that in adults, so that labour in < 20 year olds may be treated similarly to that in adult women, and that decisions about operative intervention should not be based on age alone.

However, despite the findings of other studies, we found a high incidence of postpartum haemorrhage and of manual or instrumental uterine revision in the teenage group compared with the young adult group.

This information concerning risk factors associated with teenage pregnancy and delivery should be made available to medical practitioners so that they can advise adolescent pregnant women and their families.

## Figures and Tables

**Table 1:** Maternal age distribution in the entire cohort of singleton adolescent women.

Age	12	13	14	15	16	17	18	19
Number of cases	4	9	37	145	371	710	1165	1450

**Table 2:** Risk of pregnancy comorbidities among teenagers in comparison with young adults (20–24 years of age).

Comorbidity; ICD-10 RR [CI] *p*	Age 12–17 *N* = 1276	Age 18-19 *N* = 2615	Age 12–19 *N* = 3891	Age 20–24 *N* = 9479
Age of the study group (mean ± SD)	16.35 ± 0.88	18.55 ± 0.497	17.81 ± 1.25	22.18 ± 1.42
Chronic/gestational hypertension O10; O13 RR [CI] *p*	54 (4.23%) 1.06 [0.80–1.41] *p* = 0.64	93 (3.55%) 0.89 [0.71–1.12] *p* = 0.36	147 (3.77%) 0.95 [0.79–1.15] *p* = 0.65	375 (3.95%)
Preeclampsia O14.0-O14.1 RR [CI] *p*	5 (0.39%) 1.68 [0.64–4.45] *p* = 0.24	7 (0.54%) 1.15 [0.49–2.69] *p* = 0.82	12 (0.30%) 1.32 [0.65–2.68] *p* = 0.45	22 (0.23%)
Foetal anomalies O35 RR [CI] *p*	0	6 (0.22%) 0.77 [0.32–1.87] *p* = 0.68	6 (0.15%) 0.52 [0.21–1.27] *p* = 0.18	28 (0.29%)
Anaemia O99.0 RR [CI] *p*	873 (68.41%) 1.16 [1.12–1.21] *p* < 0.01	1722 (65.85%) 1.12 [1.08–1.16] *p* < 0.01	2595 (66.69%) 1.13 [1.10–1.17] *p* < 0.01	5557 (58.62%)
Diabetes (gestational, pregestational) O24.41-42; O24.0 RR [CI] *p*	1 (0.07%) 0.22 [0.03–1.64] *p* = 0.17	3 (0.11%) 0.32 [0.10–1.07] *p* = 0.06	4 (0.10%) 0.30 [0.10–0.84] *p* = 0.01	33 (0.34%)
Obesity O26.0 RR [CI] *p*	59 (0.39%) 0.39 [0.16–0.96] *p* = 0.04	18 (0.68%) 0.69 [0.42–1.14] *p* = 0.96	23 (0.59%) 0.59 [0.37–0.93] *p* = 0.03	94 (0.99%)
Low urinary tract infection O23.1; O86.21 RR [CI] *p*	344 (26.95%) 1.18 [1.07–1.30] *p* < 0.01	637 (24.35%) 1.06 [0.99–1.15] *p* = 0.09	981 (25.21%) 1.10 [1.03–1.18] *p* < 0.01	2159 (22.77%)
High urinary tract infection O23.0; O86.2.2 RR [CI] *p*	1 (0.07%) 0.23 [0.03–1.75] *p* = 0.17	4 (0.15%) 0.46 [0.16–1.32] *p* = 0.21	5 (0.12%) 0.39 [0.15–1.02] *p* = 0.06	31 (0.32%)
Pediculosis B85 RR [CI] *p*	78 (6.11%) 3.76 [2.88–4.90] *p* < 0.01	75 (2.86%) 1.76 [1.34–2.31] *p* < 0.01	153 (3.93%) 2.42 [1.90–3.00] *p* < 0.01	154 (1.62%)
Anogenital condyloma A63.0 RR [CI] *p*	24 (1.88%) 2.40 [1.52–3.80] *p* < 0.01	21 (0.80%) 1.02 [0.63–1.66] *p* = 0.90	45 (1.15%) 1.50 [1.04–2.17] *p* = 0.03	74 (0.78%)
Lues/syphilis O98.1 RR [CI] *p*	7 (0.54%) 1.52 [0.67–3.44] *p* = 0.32	16 (0.61%) 1.70 [0.94–3.08] *p* = 0.08	23 (0.59%) 1.66 [0.98–2.83] *p* = 0.06	34 (0.35%)
Trichomonas A59.0 RR [CI] *p*	12 (0.94%) 1.81 [0.97–3.40] *p* = 0.07	23 (0.87%) 1.70 [1.03–2.78] *p* = 0.04	35 (0.89%) 1.74 [1.12–2.68] *p* = 0.02	49 (0.51%)
*E. coli* B96.2 RR [CI] *p*	192 (15.04%) 1.25 [1.09–1.45] *p* < 0.01	311 (11.89%) 0.99 [0.88–1.11] *p* = 0.94	503 (12.92%) 1.08 [0.98–1.10] *p* = 0.12	1133 (11.95%)
*Streptococcus* B95.0; B95.1 RR [CI] *p*	54 (4.23%) 0.89 [0.68–1.18] *p* = 0.48	107 (4.09%) 0.85 [0.69–1.04] *p* = 0.12	161 (4.13%) 0.87 [0.73–1.04] *p* = 0.15	467 (4.92%)
*Staphylococcus* B95.6-8 RR [CI] *p*	89 (6.97%) 1.04 [0.84–1.29] *p* = 0.67	139 (5.31%) 0.78 [0.65–0.95] *p* < 0.01	228 (5.85%) 0.87 [0.75–1.01] *p* = 0.08	671 (7.07%)
*Klebsiella* B96.1 RR [CI] *p*	23 (1.80%) 1.68 [1.07–2.64] *p* = 0.02	18 (0.68%) 0.64 [0.39–1.06] *p* = 0.09	41 (1.05%) 0.98 [0.68–1.41] *p* = 0.99	102 (1.07%)
*Candida* B37.3 RR [CI] *P*	18 (1.41%) 1.81 [1.08–3.03] *p* = 0.03	19 (0.72%) 0.93 [0.56–1.54] *p* = 0.89	37 (0.95%) 1.22 [0.82–1.81] *p* = 0.34	74 (0.78%)
*Gardnerella vaginalis* B96.89 RR [CI] *p*	0	2 (0.07%) 1.20 [0.24–5.90] *p* = 0.68	2 (0.05%) 0.81 [0.16–4.02] *p* = 0.98	6 (0.06%)
HIV infection O98.7 RR [CI] *p*	2 (0.15%) 0.74 [0.17–3.17] *p* = 0.99	4 (0.15%) 0.72 [0.24–2.11] *p* = 0.80	6 (0.15%) 0.73 [0.29–1.81] *p* = 0.66	20 (0.21%)
Hepatitis B and hepatitis C B18.0-2 RR [CI] *p*	3 (0.23%) 0.22 [0.07–0.69] *p* < 0.01	15 (0.57%) 0.53 [0.31–0.92] *p* < 0.01	18 (0.46%) 0.43 [0.26–0.71] *p* < 0.01	101 (1.06%)
Premature rupture of membranes O42 RR [CI] *p*	261 (20.45%) 0.75 [0.67–0.84] *p* < 0.01	702 (26.84%) 0.98 [0.91–1.05] *p* = 0.70	963 (24.74%) 0.90 [0.85–0.96] *p* = 0.03	2581 (27.22%)
Chorioamnionitis O41.1 RR [CI] *p*	16 (1.25%) 0.47 [0.28–0.78] *p* < 0.01	73 (2.79%) 1.06 [0.82–1.37] *p* = 0.63	89 (2.28%) 0.87 [0.68–1.10] *p* = 0.27	249 (2.62%)
Placenta praevia O44.0-1 RR [CI] *p*	9 (0.70%) 0.22 [0.11–0.43] *p* < 0.01	27 (1.03%) 0.32 [0.22–0.48] *p* < 0.01	36 (0.92%) 0.29 [0.20–0.41] *p* < 0.01	297 (3.13%)

**Table 3:** Delivery characteristics and outcome among teenagers in comparison with young adults (20–24 years of age).

Delivery characteristics	Age 12–17 *N* = 1273	Age 18-19 *N* = 2615	Age ≤ 19 *N* = 3891	Age 20–24 *N* = 9479
Gestational age (M ± SD) *p*	38.13 ± 2.10 *p* < 0.01	38.39 ± 1.90 *p* = 0.38	38.31 ± 1.97 *p* < 0.01	38.42 ± 1.81
Preterm birth O60 RR [CI] *p*				
<37 wa^*∗*^ but >24 wa^*∗*^	170 (13.32%) 1.56 [1.34–1.82] *p* < 0.01	244 (9.33%) 1.05 [0.91–1.20] *p* = 0.48	414 (10.63%) 1.21 [1.08–1.35] *p* < 0.01	842 (8.88%)
<34 wa^*∗*^ but >24 wa^*∗*^	51 (3.99%) 1.68 [1.24–2.26] *p* < 0.01	70 (2.67%) 1.12 [0.86–1.46] *p* = 0.39	121 (3.10%) 1.32 [1.06–1.64] *p* = 0.01	255 (2.69%)
<28 wa^*∗*^ but >24 wa^*∗*^	5 (0.39%) 1.32 [0.51–3.42] *p* = 0.58	10 (0.38%) 1.29 [0.62–2.66] *p* = 0.43	15 (0.38%) 1.30 [0.69–2.44] *p* = 0.40	43 (0.45%)
Birth weight at delivery (gr) (M ± SD) *p*	3010 25 ± 5.33 *p* < 0.01	3136 78 ± 5.36 *p* < 0.01	3096 40 ± 538.70 *p* < 0.01	3227.08 ± 539.11
Very low birth weight <1500 g RR [CI] *p*	23 (1.80%) 1.46 [0.93–2.27] *p* = 0.11	32 (1.22%) 0.99 [0.67–1.46] *p* = 0.99	55 (1.41%) 1.16 [0.84–1.59] *p* = 0.35	117 (1.23%)
Low birth weight <2500 g RR [CI] *p*	161 (12.61%) 1.81 [1.54–2.13] *p* < 0.01	222 (8.48%) 1.21 [1.05–1.41] *p* < 0.01	383 (9.84%) 1.43 [1.26–1.61] *p* < 0.01	1043 (11.00%)
FGR (foetal growth restriction) O36.5RR [CI] *p*	184 (14.42%) 1.38 [1.19–1.60] *p* < 0.01	352 (13.46%) 1.29 [1.15–1.44] *p* < 0.01	536 (13.77%) 1.34 [1.21–1.48] *p* < 0.01	986 (10.40%)
Caesarean section O82RR [CI] *p*	292 (22.88%) 0.74 [0.67–0.82] *p *< 0.01	604 (23.09%) 0.75 [0.69–0.81] *p *< 0.01	896 (23.02%) 0.75 [0.70–0.80] *p *< 0.01	2905 (30.64%)
Episiotomy RR [CI] *p*	466 (36.52%) 1.45 [1.34–1.57] *p* < 0.01	920 (35.18%) 1.40 [1.31–1.49] *p* < 0.01	1386 (35.62%) 1.41 [1.34–1.49] *p* < 0.01	2382 (25.12%)
Perineal rupture of 1st, 2nd degree O70.0-1RR [CI] *p*	85 (6.66%) 0.51 [0.41–0.63] *p* < 0.01	258 (9.86%) 0.75 [0.66–0.86] *p* < 0.01	343 (8.81%) 0.67 [0.60–0.75] *p* < 0.01	1232 (12.99%)
Perineal rupture of 3rd, 4th degree O70.2-3RR [CI] *p*	5 (0.39%) 1.09 [0.42–2.78] *p* = 0.80	6 (0.22%) 0.63 [0.26–1.52] *p* = 0.44	11 (0.28%) 0.78 [0.39–1.55] *p* = 0.62	34 (0.35%)
Cervical laceration O71.3RR [CI] *p*	45 (3.52%) 1.51 [1.10–2.08] *p* = 0.01	74 (2.82%) 1.21 [0.94–1.58] *p* = 0.15	119 (3.05%) 1.31 [1.05–1.64] *p* = 0.05	220 (2.32%)
Instrumental delivery (Forceps and Vidextractor) O66.5RR [CI] *p*	3 (0.23%) 0.54 [0.16–1.75] *p* = 0.47	19 (0.72%) 1.67 [0.97–2.88] *p* = 0.08	22 (0.56%) 1.30 [0.77–2.19] *p* = 0.33	41 (0.43%)
Cephalopelvic disproportion O33.1; O33.4RR [CI] *p*	86 (6.73%) 1.60 [1.28–2.00] *p* < 0.01	120 (4.58%) 1.09 [0.89–1.33] *p* = 0.38	206 (5.29%) 1.26 [1.07–1.48] *p* < 0.01	397 (4.18%)
Dystocic presentations, humeral O32.2RR [CI] *p*	7 (0.54%) 2.16 [0.93–5.11] *p* = 0.08	7 (0.26%) 1.05 [0.45–2.45] *p* = 0.82	14 (0.35%) 1.42 [0.73–2.74] *p* = 0.28	24 (0.25%)
Dystocic presentations Breech O32.1 RR [CI] *p*	43 (3.36%) 0.74 [0.54–1.01] *p* = 0.06	102 (3.90%) 0.85 [0.69–1.06] *p* = 0.17	145 (3.72%) 0.82 [0.68–0.98] *p* = 0.03	430 (4.53%)
Postpartum haemorrhage O72 RR [CI] *p*	125 (9.79%) 1.60 [1.33–1.92] *p* < 0.01	214 (8.18%) 1.33 [1.15–1.55] *p* < 0.01	339 (8.71%) 1.42 [1.25–1.62] *p* < 0.01	579 (6.10%)
Instrumental/manual uterine revision RR [CI] *p*	243 (19.4%) 1.46 [1.29–1.65] *p* < 0.01	406 (15.52%) 1.19 [1.07–1.32] *p* < 0.01	649 (16.67%) 1.28 [1.17–1.40] *p* < 0.01	8247 (88.90%)
Apgar <7 at 1 min RR [CI] *p*	92 (7.21%) 1.77 [1.42–2.20] *p* < 0.01	131 (5.00%) 1.23 [1.01–1.49] *p* = 0.03	223 (5.73%) 1.40 [1.19–1.65] *p* < 0.01	386 (4.07%)
Apgar <7 at 5 min RR [CI] *p*	30 (2.35%) 2.06 [1.38–3.07] *p* < 0.01	31 (1.18%) 1.04 [0.69–1.54] *p* = 0.83	61 (1.56%) 1.37 [1.00–1.87] *p* = 0.04	108 (1.13%)
Hospitalisation (number of days) (M ± SD ) *p*	5.97 ± 3.96 *p* < 0.01	6.68 ± 3.84 *p* = 0.33	.44 ± 3.89 *p* = 0.06	6.59 ± 4.23

Notes: ^*∗*^wa = weeks of amenorrhea.
